# Neurologic and Thrombotic Complications in the Setting of Chronic Nitrous Oxide Abuse

**DOI:** 10.1155/2023/5058771

**Published:** 2023-01-13

**Authors:** Erin Meier, Monica Malviya, Sukhdeep Kaur, Jacklynn Ibrahim, Andrew Corrigan, Andrew Moawad, Sathya Alekhya Bukkuri, Joshua Trebach, Mark K. Su, Manju Pillai

**Affiliations:** ^1^Department of Pulmonary & Critical Care Medicine, NYU Langone Hospital–Long Island, Mineola, New York, 222 Station Plaza Suite 400, NY 11501, USA; ^2^Department of Internal Medicine, NYU Langone Hospital–Long Island, Mineola, New York, 222 Station Plaza Suite 500, NY 11501, USA; ^3^Division of Medical Toxicology, Department of Emergency Medicine, University of Iowa Hospitals and Clinics, Iowa City, IA, USA; ^4^New York City Poison Control Center, Department of Health and Mental Hygiene, NY, 550 1st Ave, New York, NY 10016, USA

## Abstract

Nitrous oxide is a commonly used inhaled anesthetic for medical procedures, as well as a drug of abuse throughout the world. Excessive nitrous oxide inhalation has been shown to cause a functional vitamin B12 deficiency and hyperhomocysteinemia, which can lead to peripheral neuropathy and hypercoagulability, respectively. While the development of neurologic toxicity from chronic nitrous oxide abuse (i.e., encephalopathy, myelopathy, and neuropathy) has been previously described, the thrombotic potential of chronic nitrous oxide abuse is less known. The authors report two cases of nitrous oxide abuse leading to both neurologic and thrombotic complications.

## 1. Introduction

Inhaled nitrous oxide (N_2_O), also known colloquially as “whippets,” was traditionally used in the medical and dental fields for its anesthetic effects. It was first described by Humphry Davy in 1800, and at that time, the drug had already been widely used recreationally [1]. More than 200 hundred years later, the lifetime prevalence of nitrous oxide use was found to be 23.5% of over 120,000 survey responders from more than 30 countries according to the Global Drug Survey [2]. Nitrous oxide abuse has also become highly prevalent in the younger populations because of its euphoric and antianxiety effects [3].

In addition to its euphoric effects, inhaled nitrous oxide can classically cause neurologic symptoms (e.g., numbness) by causing a functional B12 deficiency. Rarely, inhaled nitrous oxide can also lead to hypercoagulability, believed to be secondary to hyperhomocysteinemia. In this case series, we report two patients who developed both neurologic and thrombotic complications in the setting of chronic nitrous oxide abuse.

## 2. Case Presentations

### 2.1. Case 1

**Figure 1:**
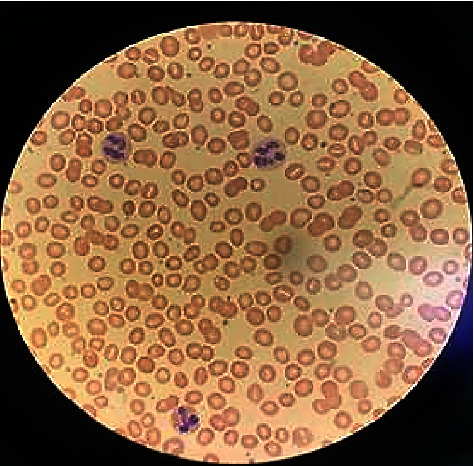
Peripheral smear showing hypersegmented neutrophils consistent with megaloblastic anemia from vitamin B12 deficiency.

**Figure 2:**
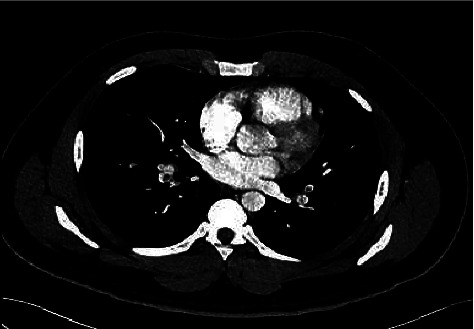
Extensive bilateral pulmonary emboli seen on chest CT angiogram.

A 22-year-old healthy man with no significant past medical history presented to the emergency department with lower extremity swelling and was found to have a deep vein thrombosis (DVT) in his right popliteal, tibial, and peroneal veins. He was discharged home on rivaroxaban. He presented to the emergency department again two days later with a chief complaint of right groin pain and ataxia. His ataxia started one week prior to presentation, along with frequent falls, bilateral foot numbness, and urinary retention. He has endorsed daily nitrous oxide “whippets” use for the last two years, using multiple canisters in a day. On physical examination, his heart rate was high at 120 beats per minute. On neurologic examination, he was found to have proximal muscle weakness in the lower extremities with 3/5 strength bilaterally and 3+ hyperreflexia of the patellar and plantar tendons. Laboratory studies revealed a megaloblastic anemia with a hemoglobin level of 10.6 g/dL (normal 13.7–17.5 g/dL), an MCV of 100 fl (normal 79.0–92.0 fl), a homocysteine level of 67.9 micromol/liter (normal less than 16 micromol/liter), methylmalonic acid of 2.417 micromol/liter, and vitamin B12 of 187 pg/mL (normal 213–816 pg/mL). Peripheral smear showed hypersegmented neutrophils (Figure 1). Computed tomography (CT) angiography showed extensive bilateral pulmonary embolism involving the right middle, right lower, left upper, and left lower lobar pulmonary arteries (Figure 2). Magnetic resonance imaging of the thoracic spine with contrast was performed, and the axial T-2 weighted images suggested areas of cord signal abnormality, but this was likely artifactual. An extensive evaluation of hypercoagulability was performed: negative prothrombin mutation, Factor-V Leiden mutation, rheumatoid factor (RF), ANA, anti-dsDNA, anticardiolipin antibodies, anti-B2gp1 antibody, and normal antithrombin III, protein C, and protein S levels. The patient had a heterozygous mutation for methylene tetrahydrofolate reductase of the C677T genotype. This genotype is not thought to be associated with excess thrombotic risk; however, actual risk may be modified by synergistic interactions with other factors. He was treated with rivaroxaban 20 mg daily, intramuscular vitamin B12 1,000 mcg weekly, and oral vitamin B12 500 mcg daily. At his two-month follow-up visit, he had stopped using nitrous oxide, had normal gait and strength, and had a vitamin B12 level of 1649 pg/mL. He was subsequently lost to follow-up.

### 2.2. Case 2

**Figure 3:**
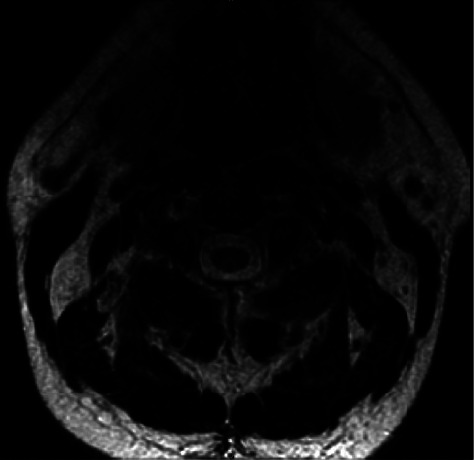
MRI of the cervical spine showing abnormal signal in the posterior columns.

A 38-year-old man with a past medical history of seizure disorder presented to the emergency department with one day of acute shortness of breath. He admitted to long term use of nitrous oxide abuse but could not quantify his use. The night prior to presentation, he reported using 25 cartridges of nitrous oxide. On presentation, he complained of bilateral lower extremity paresthesias, urinary retention, and low back pain; however, he also reported falling down a flight of stairs two months prior. In the emergency department, the patient's initial vital signs were as follows: blood pressure, 133/92 mmHg; heart rate, 117 beats/minute; respiratory rate, 22 breaths/minute; temperature, 37.3 C oral, and O_2_ Sat 99% on RA. His laboratory studies were significant for megaloblastic anemia with a hemoglobin level of 10.2 g/dL (normal 13.7–17.5 g/dL), MCV of 105.8 fl (normal 79.0–92.0 fl), vitamin B12 of less than 146 pg/mL (normal 213–816 pg/mL), a methylmalonic acid level of 8.92 *μ*mol/L (0.00–0.40 *μ*mol/L), and a homocysteine level of 127 *μ*mol/L (normal less than 16 *μ*mol/L). COVID-19 PCR was negative. The patient did not undergo any additional hypercoagulability workup, as it was presumed that his venous thromboembolisms were due to his chronic nitrous oxide use. A lower extremity venous duplex showed DVTs of the left popliteal vein and right popliteal vein extending into the right distal superficial femoral vein. A CT angiography showed bilateral central pulmonary emboli with right ventricular septal flattening and he was started on a heparin infusion. Magnetic resonance imaging (MRI) of the cervical, thoracic, and lumbar spine was performed and revealed an abnormal signal in the posterior columns of the cervical cord and an abnormal signal, more centrally located, in the thoracic cord (Figure 3). On discharge, he was continued on rivaroxaban 20 mg daily, methionine 1 g three times per day, folic acid 1 mg daily, oral vitamin B12 1,000 mcg daily, and intramuscular vitamin B12 1,000 mcg weekly, which were discontinued after three months. The homocysteine level decreased to 14 *μ*mol/L on day 7, and at 2-month follow-up, it was 10 *μ*mol/L.

## 3. Discussion

Nitrous oxide, also known as “laughing gas,” is used medically for its analgesic and sedative properties [2]. Its rapid onset, short duration, and ability to cause euphoria make it an appealing recreational drug of abuse. Inhalation of nitrous oxide can lead to death due to simple asphyxia [3]. With prolonged and heavy use of nitrous oxide, significant neurologic sequelae may manifest secondary to a vitamin B12 deficiency. Clinical effects of this functional vitamin B12 deficiency include numbness, weakness, ataxia, polyneuropathy, bone marrow suppression, and megaloblastic anemia. Although rare, nitrous oxide abuse can also be associated with a hypercoagulable state, secondary to hyperhomocysteinemia. Here, we describe two cases of chronic nitrous oxide abuse that presented with hyperhomocysteinemia, greater than four times the upper limit of normal, and vitamin B12 deficiency. Both patients were found to have neurologic and hematologic pathologies.

Nitrous oxide inactivates vitamin B12 through oxidation of the cobalt moiety. This leads to indirect irreversible inhibition of methionine synthase, which is an enzyme that converts homocysteine to methionine [2]. When inhibited, homocysteine accumulates and leads to hyperhomocysteinemia. Homocysteine is an amino acid not supplied by the diet that, when elevated, has been associated with various cardiovascular, cerebrovascular, and thromboembolic diseases [4].

Nitrous oxide abuse has been associated with thromboembolic diseases, such as pulmonary embolism (PE), deep vein thrombosis (DVT), and aortic arch thrombus. In all of these cases, the patients had hyperhomocysteinemia [5, 6]. Hyperhomocysteinemia can be caused by genetic polymorphisms, such as the thermolabile variant of methylene tetrahydrofolate reductase, hypothyroidism, and cigarette smoking [7, 8]. Both of our patients were cigarette smokers, potentially contributing to the observed hyperhomocysteinemia. Our patient in the first case underwent an extensive hypercoaguability workup, which was unrevealing. This presentation was years prior to the SARS-CoV-2 pandemic; therefore, SARS-CoV-2 was not considered as a cause for hypercoaguability. When investigating the etiology of hypercoaguability, it is important to evaluate for conditions such as Factor-V Leiden thrombophilia, the presence of prothrombin gene mutations, antiphospholipid antibody syndrome, and antithrombin III, protein C, and protein S deficiencies [9]. The patient in case two did not undergo additional hypercoaguability workup, as it was postulated that the patient's hyperhomocysteinemia was the cause of his provoked venous thromboses.

With cessation of nitrous oxide use, supplemental vitamin B12, and anticoagulation, both patients made meaningful recoveries with normalization of homocysteine and vitamin B12 levels. We postulate that chronic nitrous oxide abuse led to vitamin B12 deficiency and hyperhomocysteinemia and ultimately the occurrence of both neurologic effects and pulmonary embolism in these patients.
